# Systemic lupus erythematosus and COVID-19 during pregnancy

**DOI:** 10.1177/09612033211002270

**Published:** 2021-03-14

**Authors:** Hieronymus TW Smeele, Luis F Perez-Garcia, Koen Grimminck, Sam Schoenmakers, Annemarie GMGJ Mulders, Radboud JEM Dolhain

**Affiliations:** 1 Department of Rheumatology, Erasmus University Medical Center, Rotterdam, The Netherlands; 2 Department of Gynaecology and Obstetrics, Reiner de Graaf Gasthuis, Delft, The Netherlands; 3 Department of Gynaecology and Obstetrics, Erasmus University Medical Center, Rotterdam, The Netherlands

**Keywords:** Systemic lupus erythematosus, pregnancy, COVID-19

## Abstract

**Background:** The ongoing corona virus disease 2019 (COVID-19) pandemic is having a worldwide impact. Valuable information on the clinical characteristics of COVID-19 in pregnant patients with an autoimmune disease, such as systemic lupus erythematosus (SLE), is currently lacking. **Methods:** Herein, we describe the clinical presentation of 2 pregnant patients with SLE and mild symptomatic COVID-19 infection. **Results:** In both pregnant SLE patients, a watchful-waiting approach without initiation of treatment for COVID-19 was taken. No adverse outcomes were reported and both pregnancies resulted in healthy neonates born at term. In one patient we observed a flare in SLE disease activity, most likely attributed to discontinuing SLE treatment. **Conclusion:** Our report highlights the importance of multidisciplinary collaboration between health care professionals as well as individualized treatment decisions during unprecedented periods such as the current COVID-19 pandemic. Discontinuation of immunosuppressive drugs during the acute phase of a COVID-19 infection should be considered on a case-by-case basis. Maternal treatment decisions should be in line with current recommendations for treatment of rheumatic and musculoskeletal diseases during COVID-19 infection and in line with treatment of COVID- 19 during pregnancy.

The corona virus disease 2019 (COVID-19) pandemic has spread across the world. Infection with the novel corona virus can result in hypoxemic respiratory failure in a small percentage of patients.^1^ Compared to the SARS-CoV-1 (SARS) pandemic in 2008, which is associated with spontaneous miscarriage, preterm delivery, and intrauterine growth restriction, COVID-19 seems to have less impact on pregnancy.^2^ To date, evidence suggest that there is no increased risk of severe disease in pregnant women, with low risks of vertical transmission or fetal distress.^3–5^

Systemic lupus erythematosus (SLE) is characterized by an abnormal activation of the immune system and subsequent generation of autoantibodies resulting in a wide spectrum of systemic clinical manifestations.^6^ The clinical and therapeutic management of SLE during pregnancy is considered a clinical challenge due to the teratogenicity of effective drugs and because SLE flares are associated with a higher risk of pregnancy complications and serious adverse pregnancy outcomes.^6,7^

Currently, practical clinical information is lacking on the characteristics of COVID-19 in pregnant patients with an autoimmune disease. During the beginning of the current pandemic, we encountered 2 pregnant patients with SLE and symptomatic COVID-19. To the best of our knowledge, no reports on the presentation and outcome of pregnant SLE patients suffering from COVID-19 have been published. An overview of the cases is described in Table 1.

**Table 1.** Clinical and demographic features of the described SLE patients with COVID-19 during pregnancy and their offspring.

	Case 1	Case 2
Maternal age (years)	31	39
Disease duration (years)	16	20
Disease characteristics (SLICC/ACR score)Damage Index, organ involvement	1 (arthritis)	2 (arthritis, renal)
SLEDAI score, (description)	2 (low complement)	4 (arthritis)
Medication use	Azathioprine	Azathioprine
	Hydroxychloroquine	Etanercept
	Prednisone	
Gravidity, parity (n)	G1P0	G2P1
Mode of delivery	Induced labor	Caesarean section
Sex of the newborn	Female	Male
Gestational age (weeks, days)	38, 1	38, 5
Birthweight (grams)	2880	4205
APGAR score (after 5 minutes)	9	9
Congenital malformations	–	–

SLICC/ACR damage index indicates Systemic Lupus International Collaborating Clinics/American College of Rheumatology damage index, SLEDAI indicates Systemic Lupus Erythematosus Disease Activity Index score.

Case 1: 31 year old, gravida 1, para 0, gestational age of 38 weeks, diagnosed with SLE at age 15 and preexisting hypertension. The Systemic Lupus International Collaborating Clinics/American College of Rheumatology (SLICC/ACR) Damage Index (SDI)^8^ was 1. She presented herself at our Reproductive-Rheumatology outpatient clinic with a wish to conceive. At the time she was being treated with azathioprine (25 mg/day), hydroxychloroquine (200 mg/day), prednisone (5 mg/day). Prophylactic acetyl sialic acid was initiated after pregnancy was confirmed, this was agreed after multidisciplinary consultation. During pregnancy, SLE disease activity was mild. Based on her risk profile, she was admitted at 38 + 1 weeks of gestation to induce labor. At admission, she reported mild respiratory symptoms and tested positive for COVID-19 the next day. Her SLE treatment (azathioprine, hydroxychloroquine and prednisone) was not stopped nor reduced in dosage. She was induced on the same day as the positive test and protective measures were taken to prevent viral spreading during labor. A healthy girl with an Apgar score of 9/10, birthweight of 2880 grams, without congenital malformations was born. Vertical transmission of SARS-CoV-2 was ruled out by a negative PCR. Pathological examination of the placenta was not performed. Mother and daughter spent the night in isolation in the hospital and were discharged the next morning. The patient did not breastfeed and made a full recovery without residual symptoms.

Case 2: 39 year old patient diagnosed with SLE at age 19, SDI score 2 (lupus nephritis class 4, anti-phospholipid antibodies negative), gravida 2 para 1, 19 weeks of gestation and an obstetric history of preeclampsia. She presented herself at our Reproductive-Rheumatology outpatient clinic with a wish to conceive while being treated with hydroxychloroquine, azathioprine and etanercept. She was treated with etanercept because of disabling and therapy resistant arthritis. Prophylactic acetyl sialic acid was initiated after pregnancy was confirmed. At 19 + 0 weeks of gestation she presented herself at the emergency room with complaints of dyspnea and coughing. The diagnosis of COVID-19 was confirmed by a positive PCR test. No signs of severe disease were detected (O_2_ = 99%). Treatment with azathioprine and etanercept was discontinued. She was sent home to recover in quarantine. 9 days after the positive test, the patient reported arthralgia after which azathioprine was restarted. At day 15 after testing, she did not report any further symptoms related to COVID-19 and treatment with etanercept was restarted. Following current treatment recommendations etanercept was stopped at 30 weeks of gestation.^9^ Henceforth, oligoarthritis was confirmed by her rheumatologist (CMC1, MCP2, right knee and ankle). For this reason, she received local injections with glucocorticoids (triamcinolone acetonide). The patient was admitted to the hospital for a repeat caesarean section. A healthy son was born, Apgar score of 9/10 and a birthweight of 4205 gram. The delivery was complicated by a placenta accrete resulting in a massive hemorrhage which was treated with intravenous sulpostron, tranexamic acid, calcium en fibrinogen as well as two packed cells. Postpartum hemoglobin was 7.57 g/dl. Pathological examination of the placenta (weight: 460 gram, P33) showed no abnormalities related to a COVID-19 infection^4^: no local inflammation or fibrin depositions. Three days after delivery, the patient and her newborn were discharged from the hospital in good health.

Herein, we present two cases of pregnant patients with SLE with confirmed COVID-19 disease and mild symptoms during pregnancy. In both cases a watchful-waiting approach, in line with (inter)national guidelines, was taken without initiation of treatment for COVID-19. In both cases healthy newborns were born. One patient showed a flare in SLE disease activity, most likely induced by discontinuation of treatment and unrelated to the COVID-19 infection. Our cases highlight the importance of individualizing treatment decisions that present during unprecedented times like the current COVID-19 pandemic. Currently there is no information on how to manage COVID-19 during pregnancy in patients diagnosed with rheumatic diseases. Therefore, close collaboration between rheumatologists and gynaecologists is encouraged. The decisions whether to continue or discontinue immunosuppressive drugs should be taken using a multidisciplinary approach and should be dynamic in line with possible rapidly changing, new insights. Patients should also be informed and involved in this shared decision making process. If needed, consultation with expertise centers is advised. In mild cases, like the ones presented here, discontinuation of immunosuppressive drugs in the acute phase of COVID-19 infection should be considered on a case-by-case basis. Our approach was in line with the current European League Against Rheumatism (EULAR) recommendations for treatment of rheumatic and musculoskeletal diseases during COVID-19 infection outside pregnancy [9]. Current information on the safety of breastfeeding of COVID-19 patients is reassuring and seems to be safe.^10^ Case reports on neonatal COVID-19 are scarce and show mild disease in newborns.^10^ In the coming months, the discussion on this topic should expand and cover the safety and need of the future COVID-19 vaccine in pregnant patients with auto-immune diseases.

## Key message

An individualized and multidisciplinary therapeutic approach in pregnant SLE patients diagnosed with COVID-19 is recommended.
